# On-going collaborative priority-setting for research activity: a method of capacity building to reduce the research-practice translational gap

**DOI:** 10.1186/s12961-015-0014-y

**Published:** 2015-05-07

**Authors:** Jo Cooke, Steven Ariss, Christine Smith, Jennifer Read

**Affiliations:** NIHR Collaboration and Leadership in Applied Health Research and Care for Yorkshire and Humber (CLAHRC YH), Sheffield, South Yorkshire S10 2JF UK; STH NHS Foundation Trust, Royal Hallamshire Hospital, Sheffield, South Yorkshire S10 2JF UK; Sheffield Hallam University, Robert Winston Building, Collegiate Crescent Campus Sheffield, South Yorkshire, S10 2 BP UK; School of Health and Related Research, University of Sheffield, UK University of Sheffield, Regent Court, 30 Regent Street, Sheffield, S1 4DA UK; Barnsley Hospital Foundation Trust, Barnsley, South Yorkshire UK

## Abstract

**Background:**

International policy suggests that collaborative priority setting (CPS) between researchers and end users of research should shape the research agenda, and can increase capacity to address the research-practice translational gap. There is limited research evidence to guide how this should be done to meet the needs of dynamic healthcare systems. One-off priority setting events and time-lag between decision and action prove problematic. This study illustrates the use of CPS in a UK research collaboration called Collaboration and Leadership in Applied Health Research and Care (CLAHRC).

**Methods:**

Data were collected from a north of England CLAHRC through semi-structured interviews with 28 interviewees and a workshop of key stakeholders (n = 21) including academics, NHS clinicians, and managers. Documentary analysis of internal reports and CLAHRC annual reports for the first two and half years was also undertaken. These data were thematically coded.

**Results:**

Methods of CPS linked to the developmental phase of the CLAHRC. Early methods included pre-existing historical partnerships with on-going dialogue. Later, new platforms for on-going discussions were formed. Consensus techniques with staged project development were also used. All methods demonstrated actual or potential change in practice and services. Impact was enabled through the flexibility of research and implementation work streams; ‘matched’ funding arrangements to support alignment of priorities in partner organisations; the size of the collaboration offering a resource to meet project needs; and the length of the programme providing stability and long term relationships. Difficulties included tensions between being responsive to priorities and the possibility of ‘drift’ within project work, between academics and practice, and between service providers and commissioners in the health services. Providing protected ‘matched’ time proved difficult for some NHS managers, which put increasing work pressure on them. CPS is more time consuming than traditional approaches to project development.

**Conclusions:**

CPS can produce needs-led projects that are bedded in services using a variety of methods. Contributing factors for effective CPS include flexibility in use and type of available resources, flexible work plans, and responsive leadership. The CLAHRC model provides a translational infrastructure that enables CPS that can impact on healthcare systems.

## Background

The purpose of undertaking applied health research is to improve the health and wealth of a nation [1]. However, the connection between the development of new research knowledge and its diffusion into healthcare systems is beset with many problems. Research activity is often not translated into healthcare systems to exploit health gain. The poor connection between research and practice has been described as the second gap in translation [2]. A factor that contributes to this translational gap is that research does not adequately address service needs, nor do healthcare organisations have enough influence on shaping the research agenda [3].

The National Institute for Health Research (NIHR) in the UK has aimed to address this second translational gap through the funding of nine pilot Collaborations for Leadership in Applied Health Research and Care (CLAHRCs) as a natural experiment [4]. These cross-organisational research collaborations undertook three pillars of activity, namely i) applied research projects, ii) knowledge translation activities of getting research used in practice, and iii) increasing capacity to do and implement research. The CLAHRC’s role is to bring together universities and healthcare organisations to “*test new treatments and new ways of working*” [5] based on the needs of a specific geographical location (a region within the country). Reports on the external evaluations of the nine pilot CLAHRCs have highlighted a variation in practice undertaken to address the translational gap [6-8]. Issues such as the context, the CLAHRC’s antecedent conditions, and the social position of its leadership [6] shape such differences. Importantly, Soper et al. [7] have described the ‘flexible comprehensive’ strategies adopted by two of the CLAHRC’s they evaluated, highlighting the use of “*a range of approaches that seek to match the diverse aspects of the complex issues they face*” [7, p. 53]. Thus, due to the experimental nature of the funding call, the local and unique structures of the CLAHRCs, and the variation in need within the geographical footprint, each CLAHRC can contribute to an understanding of what works, for whom, in which circumstance in relation to the research-practice gap. This paper reflects on the lessons learned from one CLAHRC in relation to on-going collaborative research priority setting.

The concept of priority setting between users and producers of research is well recognised as a mechanism to produce useful research [9-11] and research capacity development [12]. Lansang and Dennis, for example, suggest that capacity building is an “*on-going process of empowering individuals, institutions, organizations and nations to define and prioritize problems systematically, to develop and scientifically evaluate appropriate solutions, and share and apply the knowledge generated*” [13, p. 764–5].

Prioritisation is strengthened when it is linked to resource allocation [14], and difficulties have been experienced in action research projects with no extra resource to services [15]. The CLAHRCs offer a flexible and additional source of funds and expertise to develop and support relationship building [7,8], and importantly, an opportunity to ‘follow through’ with projects where research plans are not ‘locked in’ for the full 5-year period of funding [6]. Policy documents at a global and national level suggest that priority setting processes should be ‘genuine and meaningful’ [16] to healthcare providers. If undertaken in this manner they argue that priority setting promotes trust amongst key stakeholders, stimulating interest, advocacy, and continued engagement, thus increasing opportunities for translation and innovation. It is the proposition in this paper that when priorities become translated into research activity and research action they becoming ‘meaningful’. The CLAHRCs offer an opportunity to tell the story from priority to action in a timely manner; the South-West Peninsular CLAHRC (PenCLAHRC), for example, has been able to show this [17]. They operated a priority process of question generation through a web-based interface, facilitated through locality leads and workshops. The resulting questions were then prioritised by the PenCLAHRC Executive Group, and ranked through voting by wider stakeholders. Importantly, they were able to report how this process resulted in 25 projects, which includes grant capture of £3 million. Their paper, however, does not report on whether this activity addressed the research-practice gap, that is, whether the projects changed practice or health outcomes [17]. Dubois and Graff suggest that a key question of the prioritisation process is “*Did the research provide answers that care decision makers needed*” [18, p. 2241].

Collaborative priority setting (CPS) is not an easy task. Academics and non-academics often have competing agendas and goals. McAneney et al. [19], for example, highlight that whilst University staff are often focused on publication, dissemination, and validation of findings, practitioners’ goals are more closely aligned to making an impact on practice. They argue that communication and exchange of knowledge are important factors for knowledge translation, “*it is the working collectively towards a common goal that illustrates a true conviction to translate findings and thus to discern the underlying mechanism*” [19, p. 1498].

Examples of research priority setting processes in the international literature include a range of methods, but very few examples of the impact and influence on subsequent research activity and services. A systematic review on research priority setting in low- to middle-income countries [20] has identified a range of approaches used, including discussions and forums at workshops, formal consensus methods, and ranking systems. Planning for implementation of priorities was only mentioned in one case in the review, and they conclude that a main area for improvement is to trace what happens after the priorities have been set [20].

Some specific approaches have been applied to research priority setting internationally, including that advocated by the James Lind Alliance [21-23] and the Child Health and Nutrition Research Initiative [24,25]. The James Lind Alliance approach has been used mainly in high-income countries; it adopts a staged approach including identifying key stakeholders, using the research literature and professional and user experience to identify and collate research ‘uncertainties’ which are then ranked by stakeholders, and consensus is agreed within a final workshop. Although this is a time consuming exercise it is not ‘on-going’. It produces a list of research priorities as an end product which, is it hoped, should inform and influence research funders and academia [21,23].

The Child Health and Nutrition Research Initiative is a three-staged process in which experts are asked to rank clinical outcome areas, formulate research questions linked to areas of importance, and then rank the formulated research questions in order of importance. This approach has been used in low- to middle-income countries [24,25] and its aims are to inform funders of research.

Priority setting by its very nature is context- and time-specific. This is problematic for the research-practice gap, as the usual approach to funding research requires further steps of commissioning, selection, and subsequent funding, and dissemination of research projects. This time lag is problematic to service providers in ever changing healthcare systems [3]. Therefore, at its most effective, research priority setting should be close to the decision for action, and responsive to changes in practices and services. Nuyens [11] suggests that skills and methods for undertaking priority setting and its impact on healthcare systems is a neglected area in research, and identifies a need to improve understanding.

This paper explores how the integrative model presented within the CLAHRCs [3] could offer an opportunity to establish such priority setting to promote research translation and innovation and to support CLAHRC project selection, research activity, and subsequent action into practice. Thus, it explores how one CLAHRC in the north of England used on-going CPS to address the knowledge translational gap.

### Setting

The CLAHRC under study had, at its outset, 18 collaborating organisations. These partners included two universities, 12 National Health Service (NHS) organisations including both hospital and community Trusts, and a health charity. The funding envelope for the CLAHRC was a mixture of NIHR grants with ‘matched’ funding from collaborating organisations. Match was provided as a mixed portfolio of ‘cash’ from the NHS organisation or their affiliated research charity, and match ‘in kind’, primarily through protected time for people undertaking research or knowledge mobilisation activities. The total amount of funding allocated to this CLAHRC was £20 million over a 5-year period (£10 m from NIHR and £10 as match funding).

The CLAHRC under study was developed during a relatively short time scale of 8 weeks in response to the initial NIHR call. It included 11 themes, or work streams of research and implementation activity. Initially, four themes were designed to undertake implementation activity, with the remaining seven focusing on research in clinical areas. Each theme had a ‘Theme Lead’, who was either an NHS clinician/manager or an academic, and a Theme Manager. The majority of Theme Leads held joint NHS-academic contracts or honorary contracts from either the NHS or academic sectors, mirrored by substantive contracts on the other.

The collaboration was designed to have a distributed management structure, whereby activity and decisions were made within Themes guided by a set of underlying CLAHRC principles. The coordination and leadership of the CLAHRC was undertaken by a Core Team which included a Director and Programme Manager. The Core Team also had expertise in public involvement and implementation. The CLAHRC strategy was operationalised through an Executive Committee which comprised the Core Team, Theme Leads, and Theme Managers. The working principles formed part of the strategy and were jointly agreed during an early consultative event of the CLAHRC. These were co-production, partner (organisational) engagement, addressing health inequalities, and building capacity. Co-production was described in CLAHRC induction materials as “*activity that engages the right people (service users, practitioners, NHS and care managers, and academics from a range of disciplines) to make decisions and support the conduct of projects and activities on issues that are important and matter to them*”. The principle of ‘co-production’ was of particular relevance to priority setting.

## Methods

Data for the study were collected from all 11 themes, the Core Team, and Executive Committee. Methods of data collection included semi-structured interviews and documentary evidence. This was part of an internal, formative evaluation conducted by the authors. A semi-structured interview schedule was developed through a process of theory development, based on the four principles of the programme. This paper reports on the data relating to co-production and, in particular, priority setting of projects, the mechanisms developed to do this, and expected outcomes. Twenty-seven interviews were undertaken through purposive sampling of key stakeholders at different levels of the CLAHRC (Table 1). One of these interviews was conducted with two people, resulting in a sample of 28 participants in total. This included stakeholders from a combination of different backgrounds including people who were academics, clinicians, NHS managers, or held joint appointments between the NHS and the university sector.

**Table 1:** **CLAHRC roles of research participants**

	**Interviewees**	**Cross theme workshop**
Board members	2	0
Core team	9	3
Theme participants (Theme Leads and Theme Managers)	17	15
Total	28	18

Documentary evidence included the initial CLAHRC bid, three-monthly theme activity reports that were part of the internal governance processes, CLAHRC annual reports, executive meeting minutes, newsletters, and website content for the first 2.5 years of the collaboration (October 2009 to March 2011). Additionally, notes were analysed from a cross-theme workshop (n = 18), which was undertaken to feedback the initial findings of the internal evaluation to the CLAHRC, and to explore some aspects of the findings in greater detail.

The study incorporated applied thematic analysis [26]. Interview schedules were developed from a theoretical framework of hypotheses: based on stakeholders’ assumptions about outcomes that programme activities and processes were expected to achieve. Broadly following a realist evaluation methodology [27], questions focused on outcomes that had been achieved, how these had come about (mechanisms), and the influence of specific contextual factors.

These semi-structured interviews were recorded and transcribed, and data were managed using Nvivo software (v.9). The evaluation team undertook thematic analysis, which combined exploratory [28,29], and confirmatory [30,31] approaches. All data relating to priority setting and decision-making were extracted and categorised according to initial broad topics that formed part of the analytic framework. These categorised data were then explored more deeply to discover and develop emergent themes. Themes and coding criteria were discussed amongst the evaluation team, and refined through a series of iterative stages involving regular team meetings, email communication, and individual analysis. In this way, analytical consistency was ensured, validity and reliability issues monitored, and the importance of analytical themes that cut across the entire sub-set of data could also be recognised.

Ethics approval was sought and given by the University of Sheffield, and NHS governance approval was gained from all participating NHS organisations. Informed consent was gained from all participants who were interviewed. Consent to use documentary sources of evidence was provided by the CLAHRC Core Team, Theme Leads, and Managers.

This evaluation was the product of the internal evaluation team, which might introduce bias. As with all programme evaluations, the relationships between evaluators and programme staff can be critical [32,33]. Counter to intuitive assumptions, whilst commissioning an external evaluation team can give the illusion of impartiality, often the tenuous nature of this relationship and financial dependency can lead to a less than critical approach. On the other hand, the stability afforded by being an integral part of the programme can allow a more candid approach.

External evaluations can often have difficulties with access to valuable informants and participants, which can be overcome by internal teams that have established relationships and routes of access to potential participants. This can allow close working with other programme members, resulting in a deep understanding of the programme and findings with high levels of validity [34].

Evaluations with a judgmental aim can suffer from potentially offending the subjects of the evaluation, or disrupting the organisation, and in this regard need to take care with the presentation of findings. However, this study aimed to be formative and promote the notion of continual improvement, rather than assessing the value of one person’s practice over another. This focus served to limit the deliberate positive bias that is often necessary in more judgemental investigations.

There are no set preferences regarding the use of internal or external evaluation teams, and often these distinctions are not as easy to make as one might initially assume. There can be a trade-off between getting close to the situation under investigation and attempting to maintain an appearance of impartiality (usually associated with traditional, objective scientific methods). For the purposes of this study, the methodology afforded a depth of understanding across a large and complex multi-organisational programme and the descriptive focus of the evaluation limited any conflicts of interest that might have led to systematic bias. Support from an external adviser also helped the team to enable critical reflective evaluation practice.

## Results and discussion

CLAHRC provided an environment to select and undertake projects that had real or potential impact on services. The interviews highlighted that the CLAHRC fostered appropriate contexts and supporting mechanisms to develop projects that aligned with organisational objectives, and therefore built capacity to undertake useful research with partner organisations.

Methods for opening and continuing dialogue to set priorities were evident in Themes and in the CLAHRC as a whole. Documentary data enabled the tracking of collaborative projects and highlighted when key decisions were made. The documents also highlighted how these selected projects had an impact, or potential impact, on services (see Table 2 for examples).

**Table 2:** **Methods of priority setting with examples of action and change**

**Method of priority setting**	**Description**	**Examples of projects**	**Likely area of impact on practice**
Trusted historical relationships	Discussion and on-going dialogue through contact between academics and senior managers in the Trusts, usually linked to joint academic-practice posts	Development of implementation projects linked to areas of clinical importance and quality incentives called Commissions for Quality and Innovation (CQUINs).	Improvements in patient safety and quality of care
			CQUINs target achieved with financial incentive to Trust
		Research questions to answer immediate clinical issues, e.g., poor control of young diabetics, poor attendance of young diabetics in NHS clinics	Changes in care pathways for young diabetics shaped by research
Platforms for negotiation and planning	Steering groups and strategy groups/special interest groups to develop ideas	Developing projects linked to service needs,	
		e.g., development of social marketing tools to recognise signs of stroke in Black and minority Ethnic communities	Marketing tools used in practice
	These groups include representatives from university and NHS stakeholders, many had service user representatives	Projects linked to changes in care pathway, for example, nutritional support for chronic obstructive pulmonary disease (COPD) patients	
	Some were developed as part of the CLAHRC infrastructure, whilst pre-existing platforms were co-opted by CLAHRC themes, for example, a Stroke Strategy Group	Implementing tele-care into a COPD care pathway	Changes in care pathways evident
			Health impacts on patients identified through evaluation
			Decisions not to change a pathway based on evaluation results (tele-health project)
Formal methods of consensus	Delphi and nominal group technique were used to inform projects to take to the next phase of a mental health project	Both formal processes selected projects that were undertaken in practice	Potential impact on patients and changes in care pathways if supported by findings
	Co-production workshops linked to obesity research		

### Mechanisms for collaborative priority setting

The methods of prioritisation included a range of processes, namely i) historical trusted partnerships with on-going dialogue; ii) platforms for negotiation and decision making; and iii) formal consensus methods for priority setting. The prioritisation processes varied over time and were characterized by the developmental phase of the collaboration.

#### Historical trusted partnerships with on-going dialogue

Some theme activity arose through dialogue between Theme Leads and their ongoing historical clinical links, maintained by joint posts between universities and the NHS. The initial application for CLAHRC funding was undertaken during a relatively short period of about 8 weeks. The funding call stipulated that themes should be ‘research ready’, and include projects that were geared up to start. During this initial pre-CLAHRC phase many projects were developed based on these NHS-academic historical relationships. One Theme Lead highlighted “*Ideally we should have done a lot of preparatory work but didn’t have enough time*”*.* A number of situations were described in which tacit knowledge (academic, clinical, or management) was used to set priorities. In these cases, knowledge gaps or areas for improvement shaped priorities based on a dialogue within trusted and established relationships. A Theme Lead explains:

“[The] *bid was written in great haste, and based on my experiences of delivering the health services – the priorities around that, and my knowledge around research and evaluation, that identified problems.*”

On-going historical links helped select both research and implementation projects. For example, one project focussed on improved services for young people with Type 1 diabetes. This was instigated because the Theme Lead held a joint post between a university and the NHS and knew that services demonstrated both poor attendance of young people at clinics and poor clinical outcomes (blood sugar control) in these young people. The subsequent project assessed the barriers and difficulties experienced by young people and professionals. These data were then fed back to services and used to shape a change in services through dialogue within a steering group that was subsequently set up within the CLAHRC. The evaluation of this change is currently taking place.

Another example of this type of priority setting was again developed through dialogue between a Theme Lead with a joint academic/NHS post and an NHS Executive senior manager. Through this interaction, the need for implementation projects that related to Commissioning for Quality and Innovation targets (incentive payments to NHS organisations related to quality of services) were seen to be most useful to the Trust. This was a different project than was suggested in the original CLAHRC submitted proposal and highlighted that the dialogue produced changes in project planning.

An additional example of responsiveness to emergent NHS needs occurred in the work plan focussing on using evidence in commissioning. The commissioning policy in the NHS was moving fast at the beginning of CLAHRC. As a consequence of negotiation with existing commissioner links the work plan was developed to deliver a shorter course rather than the initial timescales suggested in the original proposal, and the flexible funding envelope of the CLAHRC enabled this.

#### Platforms for negotiation

As the collaboration progressed, other methods of priority setting were developed. This included new on-going platforms for negotiation and debate, such as special interest groups, steering, and advisory groups. These were sustained as part of the matched funding arrangements, and enabled an environment to share and develop knowledge and decide some aspects of work planning.

“*X* [an NHS manger] *brought in experience from* [the NHS] *which identified deficits in the way services were designed. We came at it from experience and also written papers and reports.*”

The majority of the first year of the Collaboration was based on the development of these platforms. Some were ‘piggy backed’ on existing structures, whilst others were developed from scratch. These platforms were by far the most used method of making decisions, facilitated by the flexibility of the CLAHRC funding structure, including matched funding and the longevity of the proposed partnership.

“*In a traditional project/programme you would set everything up in advance and then work your way through it and that doesn’t really allow for anybody to influence anything that you are doing except in a very short window.*”

“[The funding] *allows us to be more flexible in responding to priorities elsewhere and finding out what works and what doesn’t.*”

Many Theme Leads and Theme Managers felt such platforms were successful, made evident by their continued support from the NHS and academics, especially during a time of austerity and financial cutbacks in public services. One Theme Manager commented that services perceived they had a say in projects without having to fund them, even though they provided a ‘match’ in the form of ‘people time’. Many CLAHRC groups continued to have good attendance, and this was perceived as a measure of value.

“[CLAHRC is] *thought to be valuable use of time* [by NHS]*,* [or they would] *vote with their feet*.”

“*The fact that they are still working with us and are keen to be engaged in new projects shows that they consider us to be good value. Because they still want to be involved, otherwise they’d drop us I’m sure*.”

#### Formal methods of priority setting

Formal methods have been used in some themes. These were often written into original research plans based on the CLAHRC brief, e.g., formal consensus techniques (nominal group technique) followed by a Delphi questionnaire in a project that developed interventions for the self-management of long term depression.

Another theme, focussing on managing and preventing obesity, developed a formal priority setting process through advice from the steering committee linked to tensions of differing stakeholder views; between academics and services, and between providers and commissioners of services. Changes in the context of the stakeholders’ landscape also contributed to this, with the shift of Public Health moving to Local Government in the UK.

The ‘Obesity’ Theme planned a series of workshops called ‘lite lunches’ designed to identify research questions for managing and preventing obesity. ‘Lite lunches’ were half day workshops including lunch, where a CLAHRC or other academic discussed the literature which related to a theoretical perspective or evaluation of a weight loss intervention, followed by one or two presentations by NHS and local government stakeholders, who discussed the service perspective using examples from practice. Issues discussed related to service delivery, practice, or commissioning services. Ideas were then generated from this process, and a report was written after each. These ideas then formed the basis of a priority setting day where decisions were made on how to progress. Two projects have commenced from this event, and progress is fed back to the Theme Steering Group.

### Enabling factors for responsive priority setting

The data highlighted enabling factors to support responsive priority setting, including flexibility of resources in the context of size and longevity of the programme, and receptive leadership.

#### Flexibility of resources

CLAHRC resources included both NIHR monies and ‘matched’ funding from partner organisations. Access to a mixed economy of matched funding (money, people’s time, infrastructure contributions), combined with the NIHR cash funds meant that Themes were able to be responsive to on-going dialogue, and could utilise these different resources to undertake projects within the programme. Flexibility was seen as beneficial as it provided opportunities to meet on-going priorities, but also reduced the risk to projects in a dynamic service environment.

“*They* [the matched people] *come to the management group and we get the benefit of their expertise and we can get them to do things … They wouldn’t do it if CLAHRC wasn’t there.*”

The longevity of the programme was also an important element of enabling trust and reciprocity. The combination of flexible resources within a long-term partnership enabled Theme project planning. It also provided opportunities for ‘pay back’ in activities linked to priorities across sectors.

“[We are] *trying to produce something that is relevant for the NHS and produces high quality publications. So everybody feels that the match is worth having. At the moment the uni is allowing it. I think it is impossible in this type of collaboration if everybody is bean counting.... We have to think about knock for knock or this feels fair*.”

Match with multiple stakeholders was seen as an advantage to ensure some sustainability in times of turmoil, to provide options across NHS organisations to conduct projects in services (through the application of matched ‘people time’ and NHS infrastructure). This was important, particularly for the university partners to ensure that research projects progressed.

Many respondents talked of the importance of aligning project objectives that were able to meet multiple organisational priorities in order to secure match. This was an important factor in making dialogue productive and impactful. These impacts included efficiency gains, improving quality of services, and producing strong academic outputs (Table 2).

“*People will work with you if it meets* [their] *objectives but don’t ask for money*.”

#### Receptive leadership

A precursor to meaningful priority setting included having leaders in the collaboration that listened and acted on what stakeholders said. This was made evident by changes in Theme activity and strategic direction. The second year Annual Report stated:

“*The co-production principle is also useful in problem identification and problem solving. For example, the Obesity Theme identified differences of opinion at the first stakeholder event described as the Big Lunch. A conclusion of this event was that the research activity, although relevant to some sectors of the NHS, was primarily driven by the academic stakeholders. Consequently the Theme’s strategic plan was reconfigured to create a Co-production work stream.*”

The outcome of this event was the development of the ‘Lite lunches’ described earlier on this paper, with subsequent continued engagement with multiple stakeholders to undertake projects that were agreed through the co-production work stream.

Another change made in the CLAHRC’s strategic direction was the distribution of research and implementation projects in Themes. The first year annual report said:

“*The original vision for the CLAHRC-XX was to have separate research and implementation themes. However, these theme categories now seem arbitrary, as many emerging projects within research themes are often categorised as implementation projects*.”

Some of this reflects a balance of activity around usefulness and immediacy of action linked to service needs and pay back. Implementation projects were designed to be immediately useful to the NHS. Research activity could then be negotiated to balance this later in the relationship.

### Benefits of a whole CLAHRC programme approach: the use of Flexibility and Sustainability Funds

On-going dialogue that shaped research and implementation activity was supported within the CLAHRC as a whole, and this was supported by additional resource that enabled action. This resource was the Flexibility and Sustainability Fund, which was distributed to Themes through the core NIHR funding. Many respondents highlighted the usefulness of these funds because it provided an on-going responsive resource that was able to ‘link to needs of clinicians and services’. Funding was used to extend the current projects, and to develop additional aspects of projects, for example, funding health economic input to answer questions about cost effectiveness (an important aspect linking to the NHS agenda). It also supported cross-Theme working, promoting added benefits across the collaboration. By the end of the third year of the collaboration, 13% of projects were cross-Theme projects linked to priorities arising from on-going dialogue. The majority of this activity linked research and implementation themes together demonstrating commitment to knowledge mobilisation into the NHS. Nine of these cross-theme projects demonstrated successful grant capture, representing a combined income of over £1 million. This, along with the added resource and expertise within the CLAHRC, has produced synergies and other funding opportunities. One Theme Lead, for example, said that they were able to meet requirements of a submitted grant by blending the access to services and implementation skills available in the CLAHRC.

“[We] *drew on CLAHRC Themes to meet the need* [of the grant specification] *… its a huge piece of CLAHRC infrastructure which was not available in the past*.”

Another Theme Lead highlighted that reviewers from a successful grant application said that links into CLAHRC were a strength of the application. In this way, the on-going nature of the collaboration and dialogue of important practice issues illustrate research capacity building and highlight when the Collaboration as a whole would feel motivated and able to respond to external calls for funding. The Flexibility and Sustainability Fund enabled preparation for such opportunities.

### Difficulties in the CPS process

The main tension identified from participants was getting the balance right between being responsive to priorities, and the possibility of ‘drift’ within Theme or project work.

It was also recognised that negotiation was time consuming.

“*You may have to go around a different path to get to the original aim. The aims stay the same, but the objectives and how you get there may change. But this is valuable learning for CLAHRC*.”

There were also some difficulties encountered between listening to services and experiencing divergent views from different NHS stakeholders. This was particularly true within on-going platforms for dialogue, for example, in research Steering Groups. The NHS is currently divided into commissioners and providers of services, and they can have differing views and priorities. Sometimes a common ground was identified and eventually consensus agreed. In other examples, different stakeholders linked with projects that most aligned with their objectives and opted out of others. Not all projects, therefore, were by mutual consensus.

Match in terms of NHS staff time was, on the whole, considered a useful mechanism for supporting on-going dialogue and engagement that worked for most people and organisations; but this was not true for all. Although some respondents saw that having CLAHRC ‘matched’ work in job descriptions and job plans legitimised and protected time to do research, this did not work for some NHS managers in some organisations, who experienced an expansion in workload without accompanying protected time to do this.

“*Well that’s me* [as a matched partner] *and it is not real at all. My general manager sees nothing, he just sees that I have more work to do.*”

“*I’m on match and nobody funds me- and really I’ve put a considerable amount of work* [in] *at* [a highly paid] *level*.”

## Conclusions

There are limitations to this evaluation. The study took place in one CLAHRC, covering a specific geographical area. Therefore, it might be argued that the findings could be unrepresentative of other settings. However, the findings correspond well with those from national CLAHRC research studies. This triangulation of findings suggests that this study has relevance beyond the initial study area. In addition, the purpose of the study was to establish what works, for whom, in what circumstances, and why. Therefore, the study adds to the body of current knowledge by demonstrating the context within which various mechanisms operate in particular ways to produce specific outcome patterns. In this respect, the findings are universally applicable to other settings where these factors are recognisable. The other potential limitation of the study is that it took place during a specific developmental stage of the CLAHRC programme. As a consequence of this element of the study design, it could be argued that the subject would benefit from further longitudinal investigations to explore longer-term effects over extended time-scales.

**Figure 1:**
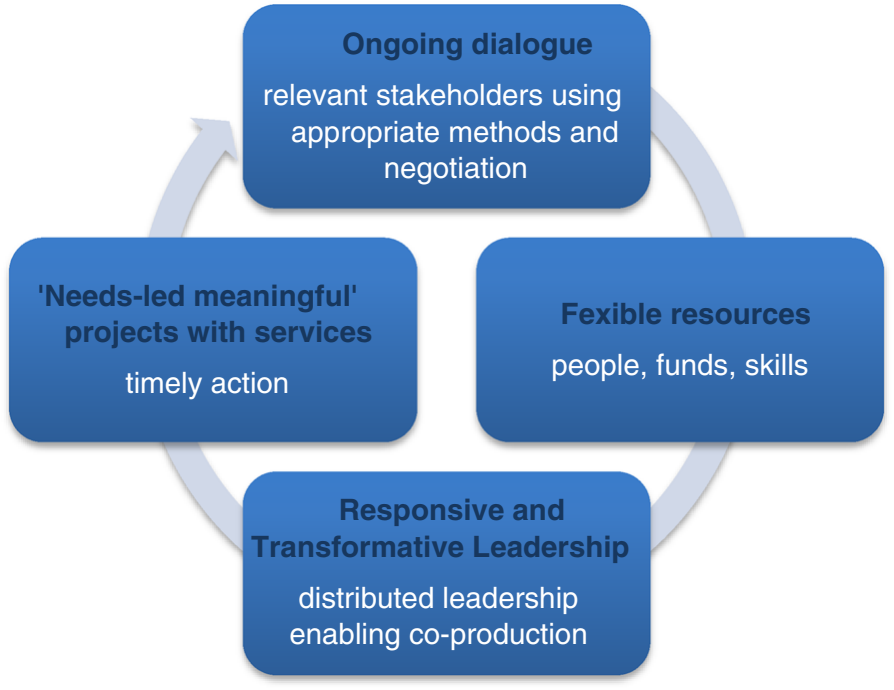
Collaborative priority setting process that builds capacity to address the translational gap.

This study examines one of the pilot CLAHRCs funded by a national funding body, the NIHR, of a high-income country (England), and commissioned to address the research-practice gap in a local context in regions of that country. Many of the research prioritisation processes described in the literature, particularly in the low- to middle-income countries [20], were designed to advocate research priorities to the national and international research funders and the research community. There is a recognition that more work is needed to adapt research priorities derived in this manner to the local context [24], and that time-lag inevitable in a staged process could lead to a disconnect between research funded and the needs of the services [3]. Nuyens [11] suggests “*while most countries have recognized that priority setting needs to be an iterative process, few have made this process a cyclical, continual one*” [11, p. 320]. This CLAHRC, as part of the NIHR experiment to address the second gap in research translation, is attempting to provide such an example. This is summarised by the diagram in Figure 1.

CPS has the capacity to impact on the translational gap by ongoing dialogue through a number of processes and methods supported by the guiding principle of co-production. This process has been aided by some unique characteristics provided by this CLAHRC, including a mixed economy of matched funding including the use of people’s time and expertise linked to cash resources. The importance of resources to support such activity has been well documented in on-going research relationships [15] and for priority setting [20], and that allocating resources (especially funds) to jointly agreed priorities engenders trust between practitioners, policymakers, and researchers [15]. Rycroft-Malone et al. [8] recognised that dedicated CLAHRC resources, including additional funding, increased the potential for engagement across academic and practice boundaries. Our study would support this observation, particularly in the use of ‘match funding’ to enable and legitimise a space for ongoing dialogue over the 5-year period, and that this space is important to support ‘co-production’ of research projects. Co-production is an important feature of the CLAHRC model [8,17,36] for creating innovative and NHS-driven projects with a potential for impact.

Our conclusion is that meaningful prioritisation requires acting upon priorities. Quite simply, participating stakeholders need to see allocation of resources to support problem solving for it to be meaningful [14]. Rycroft-Malone et al. [8] conclude at the mid stage of their evaluation of CLAHRCs, that it was unclear how resources were ‘re-positioned’ for implementation (and thus impact on services). We would suggest that this paper does describe such repositioning, and the potential for impact on services.

Responsiveness to local need, and the flexibility to respond to this, is inherent in the CLAHRC model and many examples of flexibility have been described elsewhere [6,8,35]. D’Andreta et al. [35] concluded that it is not just local context but local ‘enactment’ that can drive responsive innovation and therefore can build on the body of knowledge of what works in which circumstances. We have described how flexibility has been enacted through the distributed management structure of this CLAHRC, where leaders were linked to platforms of dialogue and negotiation. This devolved structure, linked into a flexible resource with appropriate leadership, acted as a mechanism to support responsive work, as well as the ability to respond to opportunities when they occur. Transformative leadership was encouraged by this CLAHRC’s principle of ‘co-production’. This principle could be considered the ‘brand’ of this CLAHRC. Rycroft-Malone et al. [8] suggest each CLAHRC brand acts as a mechanism of knowledge exchange and testing out of new ideas (Chief Medical Officer statement 4) [8, p. 20]. Our project reinforces the conclusions by Currie et al. [6], namely that the social position of the Theme Leader (whether NHS facing or academia facing) and the antecedent conditions (previous networks and effective networks of Theme Leaders) influenced the initial priority setting agenda, and consequently, research activity. This is manifest by the variations in practice within the themes. However, we also found that as the collaboration matured, and the leadership made the best use of resource flexibility, this enabled more responsive and creative activity.

Because relationships and dialogues were ongoing, collaborating partners could see impact and usefulness of the full story from CPS to project activity, which made the CPS a meaningful exercise. Additionally, the ‘trade off’ and balance between research and implementation projects enabled immediately useful projects for the NHS (implementation projects) from within those of a longer term nature of impact like some research projects.

Flexibility was also seen as an important mechanism to maintain programme integrity in time of service and policy change; this has also been recognized by others [7]. The ability to be flexible and responsive is supported by a unique blend of characteristics: the mixed economy of funding, the size of the collaboration enabling work to link into a number of organizations, access to a wide range of research expertise, and longevity of the funding (5 years initially). Often, the CLAHRC was seen as a stable structure in times of transition.

The literature has highlighted that priority setting is thought to be an important element of capacity building. This CLAHRC has provided examples where iterative dialogue and synergies developing between academics and practice and between disciplines within Themes, particularly blending research with knowledge mobilisation expertise, has led to increased joint theme activity and grant capture.

The notion of ‘meaningful’ priority setting through iterative and on-going research-practice relationships appears to have occurred in this CLAHRC. However, it is time consuming and some organisations find supporting protected time as ‘match’ difficult, thereby increasing the work burden on some NHS clinical and managerial colleagues who remain engaged.

The initial findings in this paper indicate that some real and some potential changes in services have resulted derived from jointly prioritised project work, but the level and type of impact on health and wealth in the area is yet to be determined, along with the sustainability of this change. The CLAHRC ‘experiment’ may provide some evidence of this as the initiative progresses.

## Abbreviations

CLAHRC: Collaboration for Leadership in Applied Research Health and Care
CPS: Collaborative priority setting
NHS: National Health Service
NIHR: National Institute for Health Research
